# Supplementary material for: Study protocol for a systematic review with meta-analysis to compare digital stress tests regarding their psychological and physiological stress responses
Source: PLoS One. 2025 Sep 26;20(9):e0331963. doi: 10.1371/journal.pone.0331963 (PMC12468937; doi:10.1371/journal.pone.0331963)
Supplement: S2 Table — (PDF) [file pone.0331963.s002.pdf]

Database	Boolean Operator	Domain according to PICO/PECO
PubMed		Participants

		Intervention/exposure and comparators
		(digital*[Title/Abstract] OR online[Title/Abstract] OR mobile[Title/Abstract] OR virtual[Title/Abstract])<br>AND<br>("acute stress"[Title/Abstract] OR "psycho* stress"[Title/Abstract] OR "stress respons*"[Title/Abstract] OR "stress react*"[Title/Abstract] OR "stress exposure*"[Title/Abstract] OR "stress detection*"[Title/Abstract] OR "stress measur*"[Title/Abstract] OR "stress intervention*"[Title/Abstract] OR "stress induc*"[Title/Abstract] OR "Trier Social Stress Test"[Title/Abstract] OR TSST[Title/Abstract] OR "social* threat*"[Title/Abstract] OR "threat* social"[Title/Abstract] OR "MIST"[Title/Abstract] OR "stroop"[Title/Abstract])<br>AND<br>(protocol[Title/Abstract] OR laboratory[Title/Abstract] OR paradigm[Title/Abstract] OR experiment[Title/Abstract])
		Outcome
	AND	((cortisol[Title/Abstract] OR alpha-amylase[Title/Abstract] OR sAA[Title/Abstract] OR heart rate[Title/Abstract] OR heart rate variability[Title/Abstract] OR HPA axis[Title/Abstract] OR skin conductance[Title/Abstract] OR ecg[Title/Abstract] OR icg[Title/Abstract] OR pre-ejection period[Title/Abstract] OR ppg[Title/Abstract] OR sympathetic[Title/Abstract] OR psychophysiological[Title/Abstract])<br>OR<br>("negative affect"[Title/Abstract] OR affect[Title/Abstract] OR PANAS[Title/Abstract] OR appraisal[Title/Abstract] OR PASA[Title/Abstract] OR mood[Title/Abstract] OR self-report[Title/Abstract] OR psychological[Title/Abstract]))
