# Supplementary material for: Study protocol for a systematic review with meta-analysis to compare digital stress tests regarding their psychological and physiological stress responses
Source: PLoS One. 2025 Sep 26;20(9):e0331963. doi: 10.1371/journal.pone.0331963 (PMC12468937; doi:10.1371/journal.pone.0331963)
Supplement: S3 Table — (PDF) [file pone.0331963.s003.pdf]

Database	Combined Search String
PubMed	(digital*[Title/Abstract] OR online[Title/Abstract] OR mobile[Title/Abstract] OR virtual[Title/Abstract]) AND ("acute stress"[Title/Abstract] OR "psycho* stress"[Title/Abstract] OR "stress respons*"[Title/Abstract] OR "stress react*"[Title/Abstract] OR "stress exposure*"[Title/Abstract] OR "stress detection*"[Title/Abstract] OR "stress measur*"[Title/Abstract] OR "stress intervention*"[Title/Abstract] OR "stress induc*"[Title/Abstract] OR "Trier Social Stress Test"[Title/Abstract] OR TSST[Title/Abstract] OR "social* threat*"[Title/Abstract] OR "threat* social"[Title/Abstract] OR "MIST"[Title/Abstract] OR "stroop"[Title/Abstract]) AND (protocol[Title/Abstract] OR laboratory[Title/Abstract] OR paradigm[Title/Abstract] OR experiment[Title/Abstract]) AND ((cortisol[Title/Abstract] OR alpha-amylase[Title/Abstract] OR sAA[Title/Abstract] OR heart rate[Title/Abstract] OR heart rate variability[Title/Abstract] OR HPA axis[Title/Abstract] OR skin conductance[Title/Abstract] OR ecg[Title/Abstract] OR icg[Title/Abstract] OR pre-ejection period[Title/Abstract] OR ppg[Title/Abstract] OR sympathetic[Title/Abstract] OR psychophysiological[Title/Abstract]) OR ("negative affect"[Title/Abstract] OR affect[Title/Abstract] OR PANAS[Title/Abstract] OR appraisal[Title/Abstract] OR PASA[Title/Abstract] OR mood[Title/Abstract] OR self-report[Title/Abstract] OR psychological[Title/Abstract]))
CENTRAL	((digital* OR online OR mobile OR virtual) AND ("acute stress" OR (psycho* NEXT "stress") OR ("stress" NEXT respons*) OR ("stress" NEXT react*) OR ("stress" NEXT exposure*) OR ("stress" NEXT detection*) OR ("stress" NEXT measur*) OR ("stress" NEXT intervention*) OR ("stress" NEXT induc*) OR "Trier Social Stress Test" OR TSST OR (social* NEXT threat*) OR (threat* NEXT "social") OR MIST OR stroop) AND (protocol OR laboratory OR paradigm OR experiment)) AND ((cortisol OR alpha-amylase OR sAA OR "heart rate" OR "heart rate variability" OR "HPA axis" OR "skin conductance" OR ecg OR icg OR "pre-ejection period" OR ppg OR sympathetic OR psychophysiological) OR ("negative affect" OR affect OR PANAS OR appraisal OR PASA OR mood OR self-report OR psychological))<br><br>(searched in Title/Abstract/Keyword)
Web of Science	((digital* OR online OR mobile OR virtual) AND ("acute stress" OR "psycho* stress" OR "stress respons*" OR "stress react*" OR "stress exposure*" OR "stress detection*" OR "stress measur*" OR "stress intervention*" OR "stress induc*" OR "Trier Social Stress Test" OR TSST OR "social* threat*" OR "threat* social" OR MIST OR stroop) AND (protocol OR laboratory OR paradigm OR experiment)) AND ((cortisol OR alpha-amylase OR sAA OR "heart rate" OR "heart rate variability" OR "HPA axis" OR "skin conductance" OR ecg OR icg OR "pre-ejection period" OR ppg OR sympathetic OR psychophysiological) OR ("negative affect" OR affect OR PANAS OR appraisal OR PASA OR mood OR self-report OR psychological))<br><br>(searched in Topic which includes abstract, title, keywords plus, author keywords)

PsycINFO	AB ((digital* OR online OR mobile OR virtual) AND ("acute stress" OR "psycho* stress" OR "stress respons*" OR "stress react*" OR "stress exposure*" OR "stress detection*" OR "stress measur*" OR "stress intervention*" OR "stress induc*" OR "Trier Social Stress Test" OR TSST OR "social* threat*" OR "threat* social" OR MIST OR stroop) AND (protocol OR laboratory OR paradigm OR experiment)) AND ((cortisol OR alpha-amylase OR sAA OR "heart rate" OR "heart rate variability" OR "HPA axis" OR "skin conductance" OR ecg OR icg OR "pre-ejection period" OR ppg OR sympathetic OR psychophysiological) OR ("negative affect" OR affect OR PANAS OR appraisal OR PASA OR mood OR self-report OR psychological))
Scopus	TITLE-ABS-KEY(((digital* OR online OR mobile OR virtual) AND ("acute stress" OR "psycho* stress" OR "stress respons*" OR "stress react*" OR "stress exposure*" OR "stress detection*" OR "stress measur*" OR "stress intervention*" OR "stress induc*" OR "Trier Social Stress Test" OR TSST OR "social* threat*" OR "threat* social" OR MIST OR stroop) AND (protocol OR laboratory OR paradigm OR experiment)) AND ((cortisol OR alpha-amylase OR sAA OR "heart rate" OR "heart rate variability" OR "HPA axis" OR "skin conductance" OR ecg OR icg OR "pre-ejection period" OR ppg OR sympathetic OR psychophysiological) OR ("negative affect" OR affect OR PANAS OR appraisal OR PASA OR mood OR self-report OR psychological)))
